# A New Traceless Technique for Cosmetic Closure of Minimally Invasive Incision and Chest Tube Fixation After Uniportal Video-Assisted Thoracoscopic Surgery

**DOI:** 10.3389/fsurg.2022.874983

**Published:** 2022-06-30

**Authors:** Zihao Chen, Ning Xin, Kenan Huang, Rongqiang Wei, Chengdong Liu, Shiwen Niu, Zhifei Xu, Xinyu Ding, Hua Tang

**Affiliations:** ^1^ Department of Minimally Invasive Thoracic Surgery Center, Second Affiliated Hospital of Naval Medical University, Shanghai, China; ^2^ Department of Biobank, Second Affiliated Hospital of Naval Medical University, Shanghai, China

**Keywords:** traceless technique, cosmetic closure of minimally invasive incision, chest tube fixation, uniportal video-assisted thoracoscopic surgery, removal-free technique

## Abstract

**Background:**

With uniportal video-assisted thoracoscopic surgery (VATS) becoming mainstream, how to make the incision cosmetic has attracted much attention. This study aimed to introduce a new traceless method for cosmetic closure of the incision and a special procedure for chest tube fixation after uniportal VATS and to evaluate the feasibility, effectiveness, and safety of this new technique.

**Methods:**

In this retrospective study, a total of 258 consecutive patients who underwent uniportal VATS were included. Among them, 127 patients were treated with a conventional method, and 131 patients were treated with a new method.

**Results:**

Patients in the new method group had a significantly less incidence of subcutaneous emphysema after the chest tube was removed. The incidence of pneumothorax after the chest tube was removed and fat liquefaction of chest incision was not significantly different between the two groups. No differences in the incidence of pneumothorax after chest tube removal and fat liquefaction of postsurgical incision were found between the two groups. Additionally, there was also no significant difference in follow-up items.

**Conclusions:**

Taken together, our results showed that this new method for minimally invasive incision closure and chest tube fixation after uniportal VATS was as feasible, effective, and safe as the conventional one but more cosmetic.

## Introduction

A chest tube is used to drain the pleural space after thoracic operations, and chest drainage has been a standard procedure since the tube has been used for centuries. Although usage of a chest tube after thoracic surgery has a long history, chest tube-related complications such as pain, pneumothorax, or unaesthetic scar always haunt thoracic surgeons and patients. In recent years, thoracic surgeons have tried their best to improve the ability of chest tube management and reduce postoperative complications via focusing themselves on studies of seeking optimal ways for chest tube removal (1, 2), using chest drainage systems (3), changing the material of traditional chest tube (4), and so on. Almost all of them obtained satisfactory achievements and some even change our traditions and opinions. However, it seems that few surgeons pay attention to the method of chest tube fixation and incision closure.

Nowadays, with the conception of minimally invasive surgical technique popularized in thoracic surgery, more and more patients care about the beauty of surgical wounds, and many thoracic surgeons have also tried their best to achieve minimally invasive incision closure cosmetically (5, 6). Since Gonzalez-Rivas first reported uniportal thoracoscopy lobectomy in 2011 (7), uniportal video-assisted thoracoscopic surgery (VATS) has become the main minimally invasive surgical method for pulmonary surgery. Different from traditional multiport VATS, the chest tube usually has to be placed in a single incision before the uniportal VATS is finished. For better chest tube management and to make the incision cosmetic, we attempted a special procedure to fix the chest tube and a new traceless and full-layer suture method to close the single incision after uniportal VATS. In our center, this new method had been used since September 2018, and there were no severe complications related to it. Thus, the purpose of this retrospective study was to introduce this new technique and evaluate the surgical feasibility, effectiveness, and safety of the new technique by comparing it with the conventional method for anchoring chest tubes and suturing the uniportal incision after uniportal VATS.

## Methods

We retrospectively reviewed the records of 271 consecutive patients who had lung tumors and underwent lobectomy resection via uniportal VATS at Shanghai Changzheng Hospital between November 2018 and November 2020. Patients whose operation procedure shifted from VATS to an open thoracotomy (*n* = 7), who had a pulmonary operation before or a bilateral pulmonary resection (*n* = 2), or who had two chest tubes (*n* = 4) were excluded from the study. All the pulmonary resection via VATS was operated by three experienced thoracic surgeons. Meanwhile, the chest tubes were fixed and the chest drain incisions were sutured by the same thoracic surgeon.

All patients were given a full-body computed tomography scan and other necessary routine examinations for excluding surgical contraindications after they were admitted to the hospital. Before the operation, all patients were informed of the procedures of the operation and possible complications and signed the informed consent. The next day after the operation, a bedside chest x-ray was taken to inspect whether the lung was expanded satisfactorily. All patients underwent chest radiographs in the Department of Radiology the day after the chest tube was removed for detecting any possible complications. This study was approved by the institutional ethics committee.

### Methods of Chest Tube Fixation and Uniportal Incision Closure

**Figure 1:**
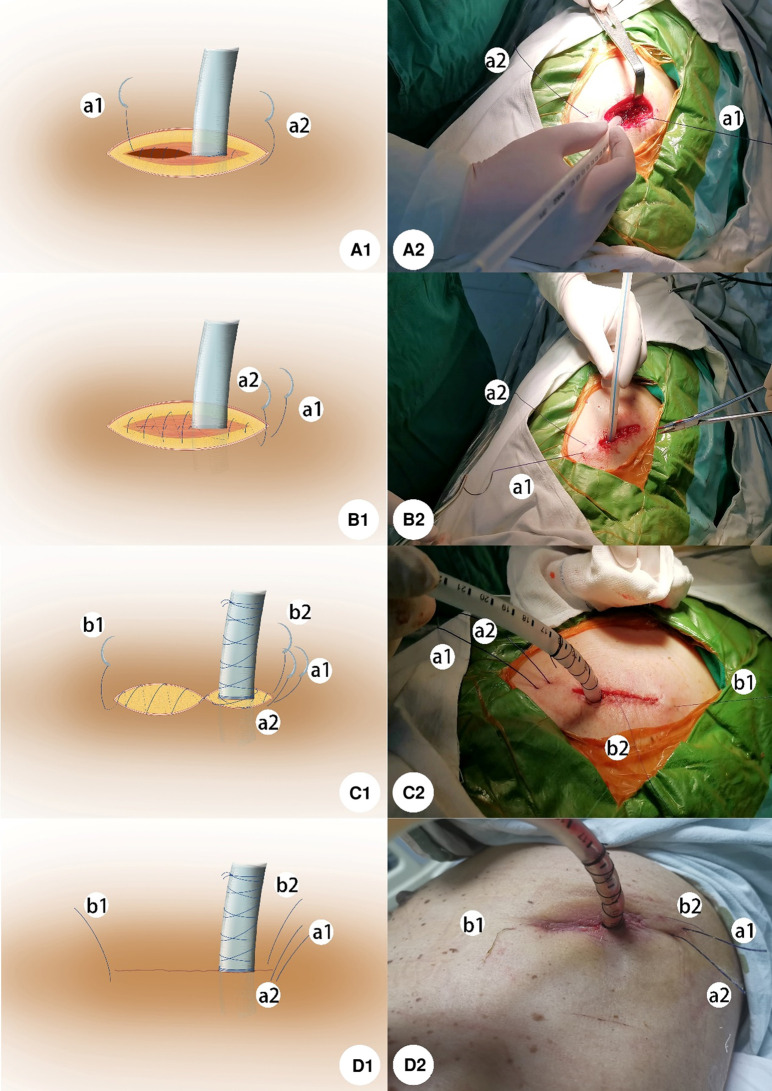
(**A1**) Suture of the muscle layer started in the middle of the incision and some knots were tied to lock up the suture. Then, the muscle was sutured continuously from the middle of the incision to each side. (**B1**) On the tube side, the side of a2 of the suture passed under the skin and came out through the skin about 1.5 cm from the edge of the incision when the needle reached the end of the incision. On the side of a1, after the suture of muscle finished, the suture of subcutaneous tissue started with a continuous suture technique by the same needle from this side to the chest tube side, and the stitch came out through the skin same as before. (**C1**) For anchoring the chest tube, a horizontal mattress suture was used to suture the dermis and some subcutaneous tissue followed by a knot in a side of the incision to tighten up the tissue around the tube. Then using the thread twined around the tube with tension in opposite directions orderly. Several rounds in the beginning enlaced at the root of the tube for stabilization, and the following rounds climbed around the tube wall up to 6–8 cm away from the tube root. For closing the dermis layer, a continuous suture was used from the middle of the incision to each side. When reaching each end of the incision, two needles came out through the skin about 1 cm away from each edge of the incision respectively. (**D1**) Procedure finished as shown in the picture. A-D2 shows the actual operation pictures corresponding to the schematic diagram of A-D1.

For this new method of chest drain wound, a 28F chest tube was inserted through the uniportal VATS incision. First, a knotless-closure suture, 5.0 metric violet monofilament synthetic absorbable PDO suture (QUILL, Wyomissing, PA), was used to suture the muscle and subcutaneous tissue beginning at the middle of the incision. This knotless-closure stitch had needles in both ends of the suture and its unidirectional anchoring barb made sure the suture would not slip back after being tightened. The suture started with a muscle layer in the middle of the incision, and some knots were tied to lock up the suture after the muscle was sutured, and then the muscle of the incision was sutured continuously from the middle of the incision to each side (Figures 1A1, A2). On the tube side, attention should be paid to protecting the chest tube from the knotless-closure stitch; meanwhile, after the needle reached the end of the incision, the suture passed under the skin and came out through the skin about 1.5 cm from the edge of the incision. On the other side, when the suture of the muscle finished, the suture of subcutaneous tissue started with the continuous suture technique by the same needle from this side to the chest tube side, and the stitch came out through the skin same as before (Figures 1B1, B2).

Second, a silk thread was used to anchor the chest tube. This novel method was to suture the dermis and some subcutaneous tissue of the wound as follows. A horizontal mattress suture was used to suture the dermis and some subcutaneous tissue followed by a knot on a side of the incision to tighten up the tissue around the tube. Then, using the two ends, the suture was twined around the tube with tension in opposite directions orderly. The several rounds in the beginning should enlace at the root of the tube for stabilization, and the following rounds needed to climb around the tube wall about 6–8 cm away from the tube root. It was important to tighten up the suture for making sure the tube anchored stably and the procedure ended with several knots (Figures 1C1, C2).

Third, a 4-0 violet monofilament synthetic absorbable PDO suture (QUILL, Wyomissing, PA) was used to close the dermis with the method of continuous suture the same as suturing muscle. However, two needles were placed horizontally through the subcutaneous tissue by passing through the opposite sides of the wound from the middle of the wound to each side. When reaching each end of the incision, two needles came out through the skin about 1 cm away from each edge of the incision respectively (Figures 1C1, C2). Finally, four needles were cut off after the whole procedure was finished (Figure 1D1, D2).

**Figure 2:**
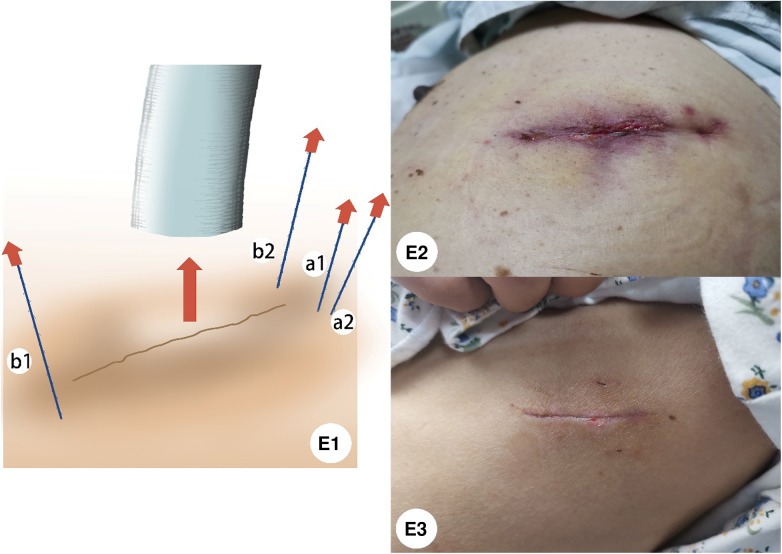
(**E1**) Before the chest tube is removed, loosen the thread twined around the tube and pull it out of the tissue. When the chest tube was removed, the incision was pressed by gauze swiftly, and then pull the rest of the knotless stitches were one by one to tighten the suture, the wound sealed like closing a zipper. (**E2**) Appearance of the incision after the chest tube is removed. (**E3**) One month after the chest tube was removed, the wound of uniportal VATS in the new method group was healed cosmetically.

The thread twined around the tube needed to be loosened and cut off before the chest tube was removed, and then the slip thread could be pulled out of the tissue. While patients were asked to keep a Valsalva maneuver at the end of full inspiration, the chest tube was withdrawn without hesitation and the incision was pressed swiftly by a gauze avoiding air from moving into the thoracic cavity. The rest of the knotless stitches were pulled one by one to tighten the suture, and the wound sealed like closing a zipper. Finally, the leftover knotless stitches were cut off and nothing was left in the skin (Figures 2E1, E2).

For the conventional method, the interrupted suture technique was used to close the muscle, subcutaneous tissue, and skin of the chest drain wound respectively after uniportal VATS in our institution. A suture beside the tube was used to anchor the tube, and the other suture was left for sealing the incision after the chest tube was withdrawn. The tube was also removed after patients kept a Valsalva maneuver at end of full inspiration, and then the wound was closed immediately with the thread left in advance by tying several knots.

The chest tube was removed when there is no active air leak and the fluid drainage was less than 200 ml. All patients were asked to keep a Valsalva maneuver during the tube removal and chest tubes were removed at the end of full inspiration. A pectoral girdle was also used for every patient to press the incision after the chest tube was removed.

### Statistical Analysis

Statistical analysis was performed using IBM SPSS version 22 (SPSS Statistics v22, IBM Co., Somers, NY, USA). The *t*-test, chi-square test, or Fisher’s exact test was used to compare the differences between the two groups. Continuous variables were expressed as the mean ± SD (standard deviation). The level of significance was defined as *p * <  0.05.

## Results

In total, 258 patients who underwent lobectomy resection via uniportal VATS between November 2018 and November 2020 were finally included in the study. Of these, 127 had sutured chest incision and anchored the chest tube with the conventional method and 131 patients were treated with the new method. As shown in Table 1, no significant differences in baseline characteristics were found between the two groups. The patients in the new method group had significantly fewer cases of subcutaneous emphysema after chest tube removal (7.9% vs. 2.3%; *p* = 0.04) (Table 2). The difference in the incidence of pneumothorax after chest tube removal was not statistically significant (2.4% vs. 1.5%; *p* = 0.68). The incidence of fat liquefaction of postsurgical incision between the two groups was also not significantly different (1.6% vs. 3.1%; *p* = 0.68). In this study, no pleural effusion leakage before chest tube removal and chest rube prolapse after the operation was reported. One patient experienced pleural effusion leakage after chest tube removal in the conventional method group and two in the modified method group. There was no significant difference between the two groups in postoperative hospitalization days, chest tube duration, or other postoperative complications.

**Table 1:** Baseline characteristics.

	Conventional method group (*n* = 127) (%)	Modified method group (*n* = 131) (%)	*p-*Value
Age (years)(±SD)	63(11.2)	63.9(11.7)	0.310
Gender			0.520
Male	71(55.9%)	68(51.9%)	
Female	56(44.1%)	63(48.1%)	
Stop smoking < 2 months before operation	26(20.5%)	32(24.4%)	0.447
BMI(±SD)	23.67(3.44)	23.69(2.88)	0.944
Hypertension	25(19.7%)	28(21.4%)	0.737
Diabetes	14(11.0%)	19(14.5%)	0.403
Oncology			0.719
Benign	4(3.1%)	3(2.3%)	
Malignant	123(96.9%)	128(97.7%)	

**Table 2:** Comparison of outcomes between two groups.

	Conventional method group (*n* = 127) (%)	Modified method group (*n* = 131) (%)	*p*-Value
Postoperative hospitalization days (±SD)	7.98 ± 3.80	7.35 ± 3.44	0.17
Chest tube duration (*d*) (±SD)	2.35 ± 1.11	2.48 ± 1.76	0.46
Pneumothorax after chest tube removal	3(2.4)	2(1.5)	0.68
Subcutaneous emphysema	10(7.9)	3(2.3)	0.04
Fat liquefaction of chest incision	2(1.6)	4(3.1)	0.68
Pleural effusion leakage before chest tube removal	0	0	0
Pleural effusion leakage after chest tube removal	1(0.8)	2(1.5)	1
Chest tube prolapse	0	0	0
Chest pain caused by the chest tube	31(24.4)	36(27.5)	0.58
Atelectasis	5(3.9)	6(4.6)	0.80
Arrhythmia	18(14.2)	22(16.8)	0.56
Pulmonary embolism	1(0.8)	0	0.49

**Figure 3:**
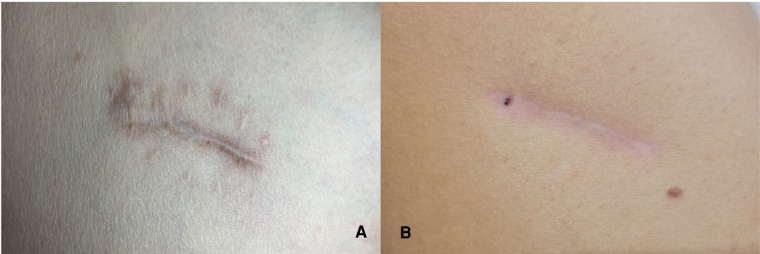
(**A**) Healing status of the wound with the conventional method 6 months after the operation; (**B**) Statement of the wound with our modified method 6 months after surgery.

The image compares the healing status of the wounds between the two methods half a year after surgery (Figure 3).

## Discussion

Recently, with the conception of minimally invasive surgery widely accepted, the invasive chest incision also has been paid more attention by thoracic surgeons and patients prefer a cosmetic wound (8, 9). As the uniportal thoracoscopic surgical technique advances, new or modified methods are required to satisfy the demands of stabilization of the chest tube and beauty of incision. Many surgeons have strived to make this technique perfect and decrease the procedure-related morbidity for patients (6, 10, 11). Xu et al. introduced a continuous suture combined with removal-free stitch for incision closure of uniportal VATS, which accomplished cosmetic closure (10). However, the dates of outcomings of the method they introduced were not provided and there might be some flaws. First, the chest tube was movable and could still insert into the thoracic cavity because the tube was anchored in the muscle layer and a knot for fixing the tube was tied about 5 cm away from the incision, which might increase the risk of infection and cause chest pain during the postoperative activity. Second, the fixed thread is still left in the subcutaneous and dermis layer after the chest tube is removed, which may hinder the healing of the incision. Third, the drainage port of the subcutaneous layer was left un-closed because the intermittent suture method was used to close the subcutaneous lay originally. Different from the method introduced by Xu et al., to avoid affecting wound healing, the incision was full-layer-sutured and the fixed thread would be completely withdrawn from the incision before the chest tube was removed in our new method. Meanwhile, the chest tube was stable and immovable with the special procedure of winding the chest tube by a thread to prevent it from coming out or inserting into the chest cavity.

In this study, all operations were performed by the same thoracic surgeon team, all chest tubes were removed with the same technique, and all patients had similar chest tube management. We believed that the lower rate of postoperative subcutaneous emphysema in the new method group has resulted from the full-layer closure of the incision after the chest tube was removed. The tissue including muscle, subcutaneous, and skin was tightened up immediately when three of the knotless threads left originally were pulled forward after chest tube removal. However, in the conventional method, it was difficult to completely close the deep muscles and subcutaneous tissues of the chest drainage incision with the thread left originally after the drainage tube was removed, which might cause the subcutaneous emphysema and pleural effusion leakage after the chest tube was removed. Although the incidence of pneumothorax after the chest tube was removed was not significantly different between the two groups, the air might move into the pleural space when sealing the chest drainage wound by tying several knots with the left thread after the chest tube was removed in conventional method group. While in the new method group, the chest drainage wound was pressed with gauze after the chest tube was removed until the tissue tightened up, which reduced the possibility of air entering the chest cavity.

Horizontal mattress sutures can tighten up the tissue, eliminate the space between the incision and chest tube wall and stop incision bleeding at the same time. However, Tomlinson and Treasure reported that horizontal mattress suture should be avoided because it converts a linear wound into a circular wound, which is unsightly (12). Fortunately, in our modified method the horizontal mattress suture was performed in the dermis and subcutaneous layer, and the thread was removed before chest tube removal. The incision with this special procedure was healed satisfactorily. Meanwhile, several loops twined around the tube in the opposite direction increased the friction force between silk and the tube wall that fixed the tube firmly and immovably. In addition, more flexibility as another achievement of this procedure should be introduced. According to clinical observation, most postoperative chest pains were related to chest drainage and the pain was relieved immediately after adjusting the depth of the tube. Therefore, when anchoring the chest tube with this procedure, it was easy to loosen the stitch and adjust the depth of the tube to relieve the chest tube-related pain. The most important thing was that there was still enough stitch to re-stabilize the tube instead of being re-sutured.

Furthermore, the incision closed with the new method was traceless and removal-free after the chest tube removed, which could prevent patients from rushing to the hospital again for removing stitches during the recuperation period after being discharged from the hospital and could also decrease doctors’ work burden.

## Conclusions

In summary, through this retrospective study, we introduced a special traceless method for minimally invasive incision closure and chest tube fixation after uniportal VATS. Meanwhile, we also showed this new method was as feasible, effective, and safe as the conventional one but made the incision more cosmetic.

## Data Availability

The original contributions presented in the study are included in the article/supplementary material, further inquiries can be directed to the corresponding author/s.
